# Climate Change and Children’s Health—A Call for Research on What Works to Protect Children

**DOI:** 10.3390/ijerph9093298

**Published:** 2012-09-10

**Authors:** Zhiwei Xu, Perry E. Sheffield, Wenbiao Hu, Hong Su, Weiwei Yu, Xin Qi, Shilu Tong

**Affiliations:** 1 School of Public Health & Institute of Health and Biomedical Innovation, Queensland University of Technology, Kelvin Grove, Brisbane, QLD 4059, Australia; Email: xu.zhiwei@student.qut.edu.au (Z.X.); weiwei.yu@qut.edu.au (W.Y.); xin.qi@student.qut.edu.au (X.Q.); 2 Department of Preventive Medicine and Pediatrics, Mount Sinai School of Medicine, New York, NY 10029, USA; Email: perry.sheffield@mssm.edu; 3 School of Population Health, University of Queensland, Brisbane, QLD 4066, Australia; Email: w.hu@sph.uq.edu.au; 4 Department of Health Statistics and Epidemiology, School of Public Health, Anhui Medical University, Hefei 230032, China; Email: suhong5151@sina.com

**Keywords:** climate change, child health, mechanism, mitigation, adaptation

## Abstract

Climate change is affecting and will increasingly influence human health and wellbeing. Children are particularly vulnerable to the impact of climate change. An extensive literature review regarding the impact of climate change on children’s health was conducted in April 2012 by searching electronic databases PubMed, Scopus, ProQuest, ScienceDirect, and Web of Science, as well as relevant websites, such as IPCC and WHO. Climate change affects children’s health through increased air pollution, more weather-related disasters, more frequent and intense heat waves, decreased water quality and quantity, food shortage and greater exposure to toxicants. As a result, children experience greater risk of mental disorders, malnutrition, infectious diseases, allergic diseases and respiratory diseases. Mitigation measures like reducing carbon pollution emissions, and adaptation measures such as early warning systems and post-disaster counseling are strongly needed. Future health research directions should focus on: (1) identifying whether climate change impacts on children will be modified by gender, age and socioeconomic status; (2) refining outcome measures of children’s vulnerability to climate change; (3) projecting children’s disease burden under climate change scenarios; (4) exploring children’s disease burden related to climate change in low-income countries; and (5) identifying the most cost-effective mitigation and adaptation actions from a children’s health perspective.

## 1. Introduction

Human-induced climate change is one of the biggest social, environmental and public health challenges we face in this century [1]. Groups such as children, the elderly and people with low socioeconomic status are particularly vulnerable to the effects resulting from such change [2]. Climate change poses a significant threat to children’s health because children have unique metabolism, behavior, physiology and development characteristics [3]. Many of the main killers of children (e.g., malaria, diarrheal disease and malnutrition) are very sensitive to climatic conditions and they are expected to worsen as a result of climate change. Changes in the spectrum of vector-borne diseases, and increasing air pollution from the continued burning of fossil fuels also threaten children’s health, quality of life, access to education and overall development [2]. Stern *et al*. reported that climate change could cause an additional 40,000 to 160,000 children’s deaths per year in South Asia and sub-Saharan Africa through gross domestic product losses alone, under a baseline climate change scenario. Under a high climate change scenario, this projection increases to an additional 60,000 to 250,000 children’s deaths per year by 2100 [4]. The World Health Organization (WHO) estimated that climate change contributed to more than 150,000 deaths and 5.5 million lost disability-adjusted life years worldwide, in 2000 alone [5], and more than 88% of this burden occurs in children under the age of five years [6,7]. 

Compared with adults, children breathe more air, drink more water, and eat more food per unit of body weight [8]. They often play outdoors, and are expected to live longer than adults, exposing them to newly developing or worsening environmental hazards in the future. Additionally, many diseases have a long latency period, sometimes requiring decades to develop. Their hand to mouth behaviour, and the fact that they live and play close to the ground further increases children's exposure to environmental hazards [9]. There has been increasing interest in climate change’s impact on children’s health [10]. Though warming in cold regions may have some beneficial effects on children (e.g., reducing some cold-related deaths and diseases, such as viral diarrheal and respiratory infections), the negative impacts of climate change on children may far outweigh the positive effects. Previous reviews have all noted children's potential increased vulnerability to climate change but there has been little discussion on the mechanisms of such vulnerability. In this article, we sought to articulate the mechanisms of children’s vulnerability to climate change (Table 1), and elucidate knowledge gaps in the existing literature. We further make some recommendations on research directions in this important field. The most common strategies to tackle climate change include mitigation and adaptation. Failure to mitigate will finally lead to the failure of adaptation because the magnitude of the impacts is predicted to become too large to manage even with considerable investment [11]. In this review, we also discuss mitigation and adaptation strategies for preventing children from being harmed by climate change in the future. 

**Table 1:** Impact of climate change on children’s health.

Impacts	Exposures	Outcomes
Direct impacts	Air pollution	Decreased lung function
		Lung cancer
		Asthma
		Birth defects
		Other respiratory disease
		Mortality
	Floods	Drowning
		Injury
		Physical and mental trauma
		Children neglect or abuse
	Droughts	Mental disorders
		Infant deaths
	Heat waves	Renal disease
		Respiratory disease
		Electrolyte imbalance
		Fever
		Possible birth defects
Indirect impacts	Decreased water	Respiratory disease
		Diarrhea
	Toxicant exposure	Reproductive disorder
		Immune dysfunction
		Neural System dysfunction
		Cancer
	Food shortage	Malnutrition
		Mortality
	Population displacement	Disrupting health care
		Loss of education
	Allergic disease	
	Infectious disease	

## 2. Methods

An extensive literature review was conducted in April 2012 using electronic databases PubMed, Scopus, ProQuest, ScienceDirect, and Web of Science. Additionally, relevant websites, such as IPCC and WHO, were also searched. Our primary search used the following U.S. National Library of Medicine’s Medical Subject Headings (MeSH terms) and keywords: *climate change, child health, risk, response, mitigation and adaptation.* Relevant empirical studies, review papers and organizational reports written in English which were published up to 1 April 2012 were included in the final assessment. Eligibility included papers or documents which considered climate change, direct and indirect environmental hazards (heat wave, weather-related disasters, and contaminated water *etc.*) resulting from climate change as the major exposure, and where children’s health was considered as one of the major outcome of interest.

## 3. Results and Discussion

We identified 1,935 scholarly papers and government and official publications in the initial search. Among which, 1,898 were primary or review papers. We firstly read the title of the 1,935 documents, and excluded 1,519 of them because of the non-relevant titles. Secondly, we read the abstracts of the remaining literature and excluded 313 of them as they did not meet the inclusion criteria. Thirdly, we read the full texts of the remaining documents and excluded 11 of them because children’s health were not specifically considered as outcome in these studies. Nine documents were added after manually inspecting the reference lists of all relevant documents. Finally, 101 documents were included. Among the remaining 101 documents, 61 were primary research, 15 were reviews, and 25 were government and official publications. 

### 3.1. Direct Impact

#### 3.1.1. Air Pollution

Air pollution is closely associated with climate change and is expected to worsen as climate change continues [12]. Climate change will affect future air quality through perturbing ventilation rates, precipitation scavenging, dry deposition, chemical production and loss rates, natural emissions, and background concentrations [13]. Climate change related wildfires could be an important source of particular matter [13]. Chang *et al*. projected the impact of climate change on ozone concentration and found that the average ozone concentration will increase by 0.43 ppb during the 2040s compared to 2000 due to climate change [14]. 

Children, together with elderly, represent subpopulations particularly sensitive to the negative health effects of air pollution [15]. Their exposure to outdoor particulate matter has been associated with respiratory symptoms, decreased lung function, worsening of asthma and the development of chronic bronchitis [8,16,17,18,19,20,21,22]. Ozone exposure may also render a number of adverse health effects in children, including shortness of breath, wheezing and coughing, temporary decreases in lung function, and lower respiratory tract infections [8,23]. Climate change negatively impacts indoor air quality too [24]. Indoor air pollution is responsible for the deaths of an estimated 1.6 million people annually, and more than half of these deaths occur among children under age five [25]. Biomass fuels use was associated with acute respiratory infections, lung cancer, tuberculosis, asthma and blindness in India. Smith found that indoor air pollution caused 4–6 per cent of the Indian national burden of disease by using a disability-adjusted life-year lost approach [26]. 

#### 3.1.2. Weather-Related Disasters

Climate change will increase the frequency and intensity of floods, droughts, bushfires and cyclones [27]. 66.5 million children, 600,000 of whom died, were affected by weather-related disasters every year from 1990 through 2000 [28]. Some studies have argued that children have more persistent symptoms of mental health impairment than adults who experience the same disaster [29]. Exposure to natural disasters was reported to exacerbate the burden of depression, anxiety and stress [30]. High rates of sleep disturbance, sadness, and other mental health impairments among children are associated with weather-related disasters [31]. In addition, high stress for adults after weather-related disasters can have serious implications for children, by impeding the adults’ ability as caregivers and in extreme circumstances resulting in neglect and even abuse [32,33]. 

Globally, floods are the most frequent weather-related disaster, accounting for 43% of all disasters between 1993 and 2001 [34]. Severe flooding in Nepal illustrated that the flood-related fatality rates for children were six times higher than mortality rates in the same villages a year before the flood [35]. In many areas of the world, drowning is a leading cause of death among young children [36], especially for boys 5 to 14 years of age [37]. Some of the effects can differentially impact girls. Indian women born during a flood in the 1970s were 19 percent less likely than men born at the same time to have attended primary school [38]. Extreme floods also led to an increase in the number of girls who were displaced from rural areas and became sex-workers in Bangladesh [39]. 

Droughts exacerbate desertification, which threatens agricultural production in most of African countries [40]. Desertification is associated with food and water insecurity, malnutrition and elevated infant mortality. In some regions, droughts and their associated famines are the most deadly weather-related disasters [41]. Children aged under six years in Ethiopia and Kenya who were born during a drought were more likely to be malnourished than those born in non-drought years. Children less than three years of age who were born in Niger in drought years were more likely to be stunted than those born in non-drought years [38]. Further, severe droughts were associated with vitamin A deficiencies in rural pre-school children in India [42]. 

#### 3.1.3. Heat Waves

Heat waves, sporadic periods of elevated temperatures outside the normal range of climate variability, occur throughout the world and are projected to become more frequent and intense in the future [43]. Studies in various countries have reported that children, especially very young children, are particularly vulnerable to heat waves [44,45,46]. Children’s renal disease is an important consequence of heat waves [46]. Exposure to extreme hot weather can induce heat-related conditions including hyperthermia and heat stress in children [47], and the renal system can be stressed or compromised by a suite of reflexive thermoregulatory, physiological, and circulatory adjustments. Respiratory diseases also increase among children during heat wave periods [48]. Knowlton and colleagues found that emergency department visits for electrolyte imbalance and heat-related illnesses increased during the 2006 California heat event among the 0–4 year age group [44]. They also assessed the impact of heat waves on hospital admissions for electrolyte imbalance, heat-related illnesses, acute renal failure and nephritis, but have not found significant effects [44]. A large increase in the number of calls for fever to National Health Service—a nurse-led helpline which provides health-related information, was seen for children 0–4 years in Greater London and South East regions of UK during a heat wave period [45]. Previous studies have reported the effect of temperature on adverse birth outcomes [49,50], which indicates that maternal exposure to heat waves may negatively influence children’s birth outcomes, even though there is no study specifically focusing on the impact of heat waves on birth outcomes to date. Some researchers reported that the adverse effects of heat waves were mainly due to the independent effects of hot temperature rather than the sustained duration of heat [51,52]. 

### 3.2. Indirect Impacts

Climate change is threatening a number of fragile ecosystems [19,53,54]. Children’s health depends on the continuous supply of various ecological services—“the conditions and processes through which natural ecosystems, and the species that make them up, sustain and fulfill human life” [55], and ecological services are underpinned by biodiversity which is also threatened by a number of climate change mechanisms. In addition rising sea levels and inundation of coastal areas, exacerbated by climate change, could render major disruption of social systems. 

#### 3.2.1. Decreased Water Quality and Quantity

Climate change alters the hydrologic cycle, melting sea ice, accelerating evaporation from sea and other surface waters, increasing the frequency and intensity of precipitation in some areas, and triggering drought in other areas [56]. Ground and surface water supplies can run short because of drought, and rising sea levels can cause salt water intrusion into groundwater. Storms and floods can compromise supplies of clean water [57,58]. As the temperatures rise, the replication of the pathogens which water carries (protozoa, bacteria and viruses) will also increase [59]. Contaminated water is the major cause of malnutrition and diarrheal disease, which remain a leading cause of death in children under five years of age [60]. 

#### 3.2.2. Food Supply Shortage

Increased evaporation dehydrates soils, and flooding salinates other arable land, diminishing agricultural area and productivity [61]. Children require three or four times more food per unit of body weight than adults and comprise the majority of the global population plagued by hunger [62,63]. Declining food production and rising food prices will exacerbate the global rates of malnutrition and stunting, and further threatening children’s health [64]. Some adverse effects of food supply shortages occurring in low- and middle-income countries extend beyond hunger and malnutrition. For example, many of Bombay’s young prostitutes are from poor rural villages in Nepal, where inadequate crop yields cause families to sacrifice one child so others may survive [65]. 

#### 3.2.3. Toxicant Exposure

As climate change continues, temperature, humidity and hydrologic cycles change, and the distribution of environmental toxicants change subsequently [66,67]. Increased precipitation and ice/snow melt in some areas may enhance wet deposition of persistent organic pollutants to aquatic and terrestrial ecosystems [68,69,70]. Regions of decreased precipitation might experience more volatilization of persistent organic pollutants and pesticides to the atmosphere. 

Rising temperature will enhance the toxicity of contaminants and increase concentrations of tropospheric ozone regionally [71]. Consequently, children may experience greater exposure to toxic substances. Exposure to a wide range of chemicals and environmental toxicants during childhood, especially chemical neurotoxicants, may affect the development, maturation, growth and function of organ systems of children [72,73,74,75]. Children are particularly vulnerable to the neurotoxic effects of lead: low levels of exposure can reduce intelligence quotient scores, and cause learning disabilities [76,77]. Methylmercury exposure is also a health hazard to the fetus in population groups that consume contaminated fish [78]. Exposure to persistent organic pollutants during early life stages may result in effects in later life [79,80]. 

#### 3.2.4. Population Displacement

Drought and severe floods are already forcing people to move more frequently [81]. Such displacement can have serious consequences for children, including fragmenting families and disrupting social networks. Disease outbreaks are more common in displaced communities [82,83]. There is some evidence that underlying chronic conditions or latent infections (such as tuberculosis) may be more likely to occur when people move into a new environment [84]. 

#### 3.2.5. Infectious Diseases

Climate change may influence the replication rates, survival and transmission of infectious disease pathogens [34]. Storms and floods may result in crop contamination and drinking water contamination which leads to illness [34]. Compared with adults, children consume a larger relative proportion of fruits and vegetables and spend a larger proportion of time outdoors, increasing climate-sensitive exposures such as pesticide residues on food and outdoor insect vectors [85]. Their relatively naive immune systems increase the risk of serious consequences from water- and food-borne diseases [86]. Every year, waterborne diseases such as cholera and other diarrheal diseases cause deaths of millions of children in the developing world, a number that will likely increase as climate changes [39]. 

Increasing temperatures, changing precipitation, and shifting climate variability may change the distribution of vector-borne infectious diseases by affecting the hosts (e.g., rodents, insects and snails) and the pathogens (e.g., bacteria, viruses and parasites) [87,88,89]. Children are especially prone to mosquito and tick bites because of often playing outside and close to the ground, where these insects gather [90]. In some regions of sub-Saharan Africa, the death rate from malaria in children aged 0 to 4 years is much greater than the death rate in the population aged over 14 years old [91,92]. A wide range of vector-borne diseases can affect young children in particular, the major health risk threats being malaria, schistosomiasis, Japanese encephalitis, and dengue hemorrhagic fever. Children, especially infants, are still in the process of physiological growth, and they are immunologically sensitive to some infectious disease pathogens [93]. If not fatal, infectious diseases can still have disabling consequences such as increased risk of hearing loss or other neurologic decrements following meningitis—a climate sensitive disease in a large swath of Africa [94]. 

#### 3.2.6. Allergic Diseases

Childhood asthma is a widespread health problem because of its epidemic prevalence [95]. Although the prevalence of childhood asthma decreased in some countries during recent years, such as Australia, it increased in a variety of countries [96]. Climate change is associated with changes in aeroallergens [97]. Pollen counts could rise due to multiple mechanisms such as increased ambient CO_2_ levels [98], increased temperature, or earlier spring seasons [99]. Elevated CO_2_ concentration often increases plant leaf biomass and carbon-to-nitrogen ratio which can affect not only pollen but also mold. Elevated ambient CO_2_ levels are associated with increased fungal spore production, a potential other asthma trigger [100]. Increased temperature can also affect spatial distribution and density of plants and fungi that produce aeroallergens [101]. Allergic disease will also likely be worsened because of interaction between climate change impacts on thunderstorms which can fractionate pollen into smaller particles; extreme precipitation events; and increasing heat-related ground-level ozone pollution [102]. The current global increase in childhood asthma can be partly explained by increased exposure to aeroallergens driven by climate change [103]. 

### 3.3. Mitigation and Adaptation Strategies for Children

Mitigation mainly includes technical and infrastructural investments, renewable energy implementation, and improving energy efficiency—all with the singular goal of reducing carbon pollution. While there are many sectors that contribute to carbon pollution, the transport sector is projected to have the fastest proportional growth in carbon pollution of any sector from 1990–2020 [104]. Promoting public transport use will be beneficial for rapidly reducing. Improving building energy efficiency, especially in places like India where two-thirds of the high-rise building stock is projected to be constructed over the coming decades, is also an important means of climate mitigation [105]. Increasing the urban tree canopy can also reduce carbon dioxide produced by fuel combustion [106]. At the individual level, some measures can be adopted such as the trifecta of reduce, reuse, and recycle as means of also increasing energy efficiency. Young athletes are more likely to be involved in outdoor activities, and their external thermal load is largely dependent on climate factors, including ambient temperature, humidity and solar radiation [107]. A variety of protective measures implemented by the local governments, such as providing air-conditioned meeting rooms and locker rooms for athletes, as well as cooling stations on or immediately next to the competition site, will be helpful for preventing athletes from heat-related health risks. 

Different from mitigation which will likely ultimately necessitate wide multinational accords, adaptation is better to be implemented at the local level [108]. Currently, report on specific adaptation measures for children is limited [109]. Sustainable economic development, reduction of poverty and wealth disparity, and improvement in baseline health, food security and education are fundamentally the best form of climate change adaptation. In terms of tackling natural disasters more specifically, effective awareness programmes in schools, homes and communities can contribute to the capacity of nations and communities where resources are limited to help children during disasters [110]. Post-disaster counseling can reduce the medium- and long-term mental health burden on children significantly [111,112]. Activation of early warning systems before heat waves is considered as a useful children-protective measure in handling heat wave hazards [7]. Childhood immunization programmes, drinking water disinfection, source water protection and sanitation are all necessary actions which protect children from possible adverse impacts of ecological change. Pediatricians can play important roles as advocates by individual example and through community participation to address the effects of climate change to create a healthier world for children worldwide [113,114]. 

### 3.4. Knowledge Gaps

Existing research gives strong evidence that climate change is influencing and will increasingly affect children’s health. Nevertheless, substantial information gaps remain regarding the risk climate change poses to children’s health and child-specific protection measures. 

#### 3.4.1. The Modifiers

Children are already known as a particularly vulnerable group to climate change, but: (1) what social factors are already in place that are potentially protective (e.g., caregiver behavior, school attendance, *etc.*)? and (2) what else can we do to protect children besides slowing climate change? is still unknown. Previous studies looking at the impact of climate change (e.g., temperature extremes) on human well-being tend to consider children under 15 years old as a whole [115], or group them such as 0–4 and 5–14 years old [46]. In fact, children are heterogeneous both physiologically and behaviorally, not all of them are equally at the same risk. Children under 1 year old are likely to be more sensitive to heat waves because of their extremely poor self-care ability and immature regulation systems [116], and children aged 5–14 years may be more exposed to air pollution because they are engage in outdoor activities more frequently [117,118]. And as such, considering children of different age groups and estimating their vulnerability to climate change hazards would be beneficial for developing targeted protection strategies. 

Similar to age, the role of school attendance in modifying the effect of climate change on children still remains to be understood, though some studies have touched a bit on this issue. Regular attendance at school is essential for children’s education and social skills, and prolonged non-attendance was associated with social isolation [119]. Globally, the proportion of children attending school varies widely particularly for secondary school attendance which is as high as approximately 90% in some high-income regions [120] and lower than 50% in some low-income regions [121]. Existing studies indicate the possible protective or risky effects of school attendance on the occurrence of different pediatric diseases known to be sensitive to climate factors. Checkley *et al*. revealed that school absence may affect the incidence of pediatric diarrhea [122]. Grech *et al*. found that the end of school was associated with a drop in pediatric asthma admission, and restarting school correlated with a sharp rise [123]. Nevertheless, to date, no study specifically examines whether school attendance protects children from being affected by climate change hazards. 

Besides school attendance, sports participation may also modify the impact of climate change on children’s health, because the exertion related to playing sports put children at an increased risk of being exposed to heat and air pollution [124,125]. The possible modifying effects of other factors, including gender, caregiver behavior, air conditioning use [126], nutritional status and vaccination status [127], also need elucidating as climate change continues. 

#### 3.4.2. The Appropriate Outcome Measures

Existing studies tend to use mortality and morbidity as the measures of the climate change impact, including total- and cause-specific mortality [128], hospital admissions [46] and emergency department visits [129]. Meanwhile, they were likely to treat deaths amongst children, adults and elderly as equally important. However, if most deaths were in the very elderly, who had only a life expectancy of a few years, the burden of climate change on human well-being would have less public health importance [130]. Using mortality and morbidity as outcome measures may underestimate the impact of climate change on children. Years of life lost (YLL) is an indicator of premature mortality that accounts for the age at which deaths occurred by giving greater weight to deaths at younger ages [131], and it might be a more appropriate measure to evaluate the impact of climate change on human health because of emphasizing the influence of early deaths on disease burden. Years of life lived with disability (YLD) takes into account disease duration, age at onset, and a disability weight reflecting the severity of disease [132]. The disease onset at very young age is more likely to cause larger years of life lived with disability when compared with onset at older age. Besides the aforementioned outcome measures, some other adverse consequences of climate change in children, such as loss of education, should also be considered, even though few studies to date have mentioned this [133]. 

#### 3.4.3. Children’s Disease Burden under Climate Change Scenarios

Extensive studies have estimated the existing impact of climate change on children’s health [134,135], and some researchers have projected the future influence of specific climate change hazards on the total population [136,137], but no study focuses on the projection of children’s disease burden under future climate change scenarios. Despite existing uncertainty, the potential exists to begin to estimate how climate change will affect children with respect to mortality and morbidity in the future. Questions to explore include: how will infectious disease in children be influenced by climate change and to what extent will climate change affect chronic disease burden of children? 

#### 3.4.4. The Situations in Low-Income Countries

In high-income countries, people aged under 18 years old constitute approximately 20 percent of the total population, but in some low-income countries universally considered more vulnerable to climate change, this young age group represents nearly 50 percent of the population [138]. Additionally, mortality rates of children under 5 are lower than crude mortality rates in some wealthy countries. However, under 5 mortality rates are much higher than adults in some poor countries [138]. Most studies regarding the impact of climate change hazards on children have been conducted in high-income or middle-income countries. Hajat *et al*. found that a high proportion of Delhi deaths (48%) during periods of high ambient temperatures occurred among children younger than 15 years old, which was much higher than the proportion in São Paulo (10%), which in turn was substantially greater than the proportion in London (1%) [115]. This finding indicates that children in low-income countries may experience greater climate change-related health risks than those in developed countries, but this area also need further investigation.

#### 3.4.5. The Most Cost-Effective Mitigation and Adaptation Strategies for Children

Currently, climate change mitigation and adaptation measures tailored for children are limited. Children should be the focus of climate change strategy planning process, which can be carried out in three stages. Firstly, the potential impacts of climate change on children should be documented. Secondly, children’s health ought to be a major focus of the planning process for either mitigation or adaptation activities. The needs and ideas to protect children’s health must be considered in this stage. Thirdly, once the plans are completed and being carried out, they should be tested to ensure that they are sufficient to address children’s health. Formulating acceptable adaptation measures for children as well as implementing cost-effectiveness analysis of these measures will not only improve the quality of adaptation efforts but also be beneficial for future climate change mitigation efforts. In the process of developing mitigation and adaptation strategies specifically focusing on children, the socioeconomic and some other characteristics of each target region should be taken in to account because different mitigation and adaptation strategies may be needed for different locations. 

## 4. Conclusions

Because of multiple age-specific characteristics, children are uniquely vulnerable to climate change. Despite some research has been done on evaluating the impacts of climate change on children’s health, more works in this field are urgently needed. Future health research directions should focus on: (1) How climate change impacts on children will be modified by gender, age and socioeconomic status; (2) what are the most appropriate outcome measures of children’s vulnerability to climate change; (3) how to project children’s disease burden under climate change scenarios; (4) what is the situation about children’s disease burden related to climate change in low-income countries; and (5) what are the specific mitigation and adaptation actions that are most cost-effective from a children’s health perspective. 
